# Assessment of antibody titers and immunity to Hepatitis B in children receiving chemotherapy

**Published:** 2012-09-22

**Authors:** A Shams Shahemabadi, F Salehi, A Hashemi, M Vakili, F Zare, N Esphandyari, S Kashanian

**Affiliations:** 1 Immunology department, Faculty of medicine, Shahid Sadoughi University of Medical Sciences and Health Services.; 2 Central laboratory of Yazd, Shahid Sadoughi University of Medical Sciences and Health Services, Yazd, Iran,; 3 Department of Pediatrics, Hematology, Oncology and Genetics Research Center, Shahid Sadoughi University of Medical Sciences and Health Services, Yazd, Iran.; 4 Community Medicine Department, Faculty of Medcine, Shahid Sadoughi University of Medical Sciences and Health Services , Yazd ,Iran.; 5 Saman Complex, Yazd,Iran.

**Keywords:** Hepatitis B, Vaccination, Immunity

## Abstract

**Background:**

There is a decrease in vaccine-specific antibody to certain vaccine-preventable diseases in children after chemotherapy, but the frequency of non-immune patients is not clear. In the present case-control study, was taken under investigation protection level to Hepatitis B infection in children 6 months after completing chemotherapy.

**Materials and Methods:**

In this study 68 patients with cancer and 68 healthy children were enrolled. Patients were 1.5 -12 years old with completed standard chemotherapy at least for 6 months. All the patients and healthy children were negative for HBsAg and HBeAg and had received Hepatitis B vaccination. IgG antibody concentrations against Hepatitis B Virus (HBV) were determined in the patients receiving chemotrapy and healthy subjects serum by ELISA method. IgG antibody titer > 10 mIU/ml was considered as baseline protective titer for preventing HBV infection.

**Results:**

Anti-HBs antibody titer in 19.12% of patients was less than 10 mIU/ml and 11.76% of the patients had borderline antibody titer (10-20 mIU/ml). In healthy subjects, 2.94% and 5.88% had antibody titer < 10 mIU/ml and 10-20 mIU/ml, respectively. According to statistical analysis, frequency of non immune subjects in children with cancer was significantly higher than those in healthy children (P-value=0.024).

**Conclusion:**

HBV vaccination post-intensive chemotherapy in the children with cancer is strongly recommended.

## Introduction

The most important method in preventing infectious diseases is Vaccination. By effective vaccination, many infectious diseases such as diphtheria, measles, mumps, poliomyelitis, rubella, and tetanus have almost been eliminated in the world. Today one of the hygienic determinants in each country is vaccine coverage which is accompanied with effective prevention and disease control resulting in surveillance raise in the community. Preventing of some cancers e.g. Hepatocellular carcinoma may be possible by Hepatitis B Virus (HBV) vaccination (1).

Chemotherapy and radiotherapy treatment is common method in children with malignancies. Children treated for cancer are immunosuppressed during treatment and for a variable period after completion of chemotherapy or radiotherapy (2). There is a reduction of vaccine-antigen specific antibody concentration after completion of chemotherapy. Therefore children after cancer therapy may become susceptible to infectious diseases (3). This data suggests that they may partially or totally lose the protection offered by the vaccines administered before the onset of cancer, and may not be able to adequately respond to vaccine stimulation during the disease itself and for a certain time after the cessation of chemotherapy (3, 4). Today recommendation for vaccination in bone marrow transplant patients is well established and validated. In these patients routine immunization protocols are recommend (,). There are few studies in revaccination of children after intensive chemotherapy. Most of them were established based on the data from limited published studies and specific cancers (7).

On the other hand, most of children with cancer still have a normally functioning immune system at the time of disease presentation (8, 9). The protective antibody against most specific vaccine antigens and T cell number are usually in normal range, but during cancer therapy the number of T cells and antibody titer usually decrease significantly in the patients. However, humoral and cellular immune responses tend to increase when drug administration moves from the intensive phase to the maintenance phase (10). Revaccination of children with Acute lymphoblastic leukemia (ALL), 6 months after stopping chemotherapy made restoration of antibody responses and achievement of normal serum immunoglobin. The administration of booster vaccines to these patients has resulted in a good response rate. Advance in treatment protocols and improvement in drug efficacy had led to significant improvement in disease-free survival. On the other hand, intensive chemotherapy resulted in marked impairment in humoral and cellular immunities that could last for up to 6 months after stopping chemotherapy (11). According to new studies, Post-chemotherapy booster vaccinations produce a strong and sustained effect in humoral immunity against vaccine-preventable infectious diseases (,). It is less known about the frequency of non-immune patients, extent and duration of humoral and cellular immune system dysfunction in the case of cancers that are treated with standard-dose chemotherapy. Moreover, there is not an approved immunization program for the patients. In this study, we investigate humoral immune response to HBV vaccination in children affected by cancer at least 6 month after chemotherapy. 

## Materials and Methods

**Human Subjects**

In this study, 68 patients (male: 33, Female: 35, age average: 6.20±2.94) were selected from Shahid Sadoughi Hospital, Yazd ,Iran from March 2011 to September of 2011 (Table I). Patients samples consisted of Acute Lymphocyte Leukemia (38 patients), Acute Myeloid Leukemia (8 patients), Hodgkin lymphoma (8 patients), Non-Hodgkin lymphoma (6 patients), Neuroblastoma malignancy (5 patients) and Lung cancer (3 patients) (Table II). Acute Lymphocyte Leukemia and Acute Myeloid Leukemia were classified as hematologic malignancy and the rest of cancers were considered as non hematologic malignancy that showed in table I. The patients had been treated successfully for pediatric hematological malignancy and solid tumors using standard chemotherapy. This study was approved by Clinical Research Ethics Committee of Shahid Sadoughi University of Medical Sciences-Yazd, Iran. All the patients were selected from at least 6 months after completion of chemotherapy treatment.

In this study also 68 healthy children were recruited from Yazd central laboratory. Healthy children did not show any systemic, immunodeficiency or metabolic diseases according to their histories. All patients and healthy children had received HBV vaccination according to Iranian Health Ministry schedule vaccination program. All individuals were negative for HBsAg and HBeAg. The patients and healthy children were matched for sex and age. Informed consents of patients and healthy children were obtained to collect 3 ml of their peripheral blood samples. The serums samples were stored in -70 C until being analyzed in one bach. By using enzyme-linked immunosorbent assay (ELISA) (DiaPlus Inc) anti-HBsAg IgG antibody were measured according to the kit constructions. The antibody concentrations were calculated using standard curves. Titers equal or more than 10 mIU/ml for each patients and healthy children were defined as protective titer and titer less than 10 mIU/ml were considered as non-immune subject to HBV infection.

**Statistical Analysis**

In this case-control study, frequency of non immune patients and healthy children were analyzed by SPSS 19 software. Differences between the frequencies in two groups were obtained by Chi-Square. P-value≤0.05 was considered as significant.

## Results

According to specific antibody assay method in healthy children, 2.94% of individuals were susceptible to HBV infection, whereas in patients group 19.1% were susceptible to HBV infection. Statistic analysis confirmed that frequency of no immune subjects in patients group was significantly higher than that of the healthy control (PV=0.024) (Table III). In this study, it was also revealed that in 8 patients (11.7%) IgG antibody titer was between 10 to 20 mIU/ml that is considered as borderline protection level to some references. In healthy subjects, 5.88% had borderline protection antibody titer which it did not show significant difference in comparison with the patients (Table III). Our results showed, that 30.88% of children with cancer after chemotherapy were susceptible to HBV infection whereas 8.82 % of the healthy children were susceptible to the infection. According to the statistic analysis, the frequency of non immune children after chemotherapy was significantly higher than healthy children without chemotherapy (P=0.017). In this study, it also revealed that out of 11 of non-immune patients (age: 8.6±2.8) were female and 10 of them (age: 7.7±2.45) were male. Although, the average of age in female patients was slightly higher than the male patients, statistical analysis did not show significant difference in age of two groups (Table IV). On the other hand, in the non immune healthy children, the average of age was 7.5±2.1 that did not show significant difference with non immune patients (Table IV). 

**Table I:** Characteristic of Subjects

Parameter		Healthy subjects	Children with cancer
Age		6.3±2.93	6.29±2.94
gender	**Male**	32	33
	**Female**	36	35

**Table II:** Distribution of patients according to diagnosis, gender and immunity to HBV infection

Type of cancer	Female	Male	Non-immune female patients (antibody titer <20mIU/ml)	Non -immune male patients (antibody titer <20mIU/ml)
Acute lymphocytic leukemia	**20**	**18**	**7**	**5**
Acute Myeloid Leukemia	**4**	**4**	**2**	**1**
Hodgkin lymphoma	**5**	**3**	**1**	**1**
Non-Hodgkin lymphoma	**3**	**3**	**1**	
Neuroblastoma	**2**	**3**	**1**	**1**
Lung cancer and other cancers	**1**	**2**		**1**
Total	**35**	**33**	**12**	**9**

**Table III:** Anti-HBsAg antibody titers in serum of subjects and immunity to HBV infection

**Antibody titer**						**Total**
**<10 mIU/ml**				**10-<20 mIU/ml**	**≥20 mIU/ml**	
**Group**	**Healthy subjects**	**Count**	2	4	62	68
		**% within group**	2.94%	5.88%	91.18%	100.0%
	**Children with cancer**	**Count**	13	8	47	68
		**% within group**	19.12%	11.76%	69.12%	100.0%

**Table IV:** Frequency of immune and nonimmune individuals based on gender and age in healthy and children with cancer

	Cancer				Healthy		P-value (nonimmune individuals)
	**number**			**Age ** **(Mean±SD)**	**number**	**Age ** **(Mean±SD)**	
group	male	nonimmune	10	7.7±2.45	2	7.1±2.12	0.018
	female	nonimmune	11	8.6±2.8	4	7.8±2.6	0.031

## Discussion

Immunity to preventable infectious diseases by vaccines in immunocompromised and patients with cancer has always been a challenge in medicine. Dose of vaccine and sustain protective immune response and immunity to infectious agents are also of concern. It did not elucidate exactly whether or not patients with cancer have a defect in their immune system, but according to recent studies after chemotherapy some features of immune system may be affected (15). Based on previous studies this is a clear and validated recommendation for revaccination patients who have undergone hematologic stem cell transplantation (16). Intensive chemotherapy is still a main treatment strategy in clinics but if is not a confirmed approach in vaccination after chemotherapy. Although some studies elucidated that humoral immune response to booster vaccination in post-chemotherapy patients were similar to healthy individuals, but there is not an approved program for booster vaccination in the patients after chemotherapy. According to Ramesh study, 67% of children who received chemotherapy and immunosuppressive drug treatment had protective level of anti-HBs antibody in their serum. In another study by Vaans et al, it was shown that antibody titer to diphtheria, pertussis, tetanus and poliomyelitis after chemotherapy in 49 children affected by Acute Lymphoblastic Leukemia (ALL) was lower than that of healthy children but these titers in most of the patients were protective. According to this study, raising the titer in antibodies was observed only in the patients who received booster vaccination. To date, It is not clear how much time is needed for immune reconstitution after intensive chemotherapy in the patients (3, 17, 18). Information about immunity to preventable infectious diseases after chemotherapy and radiotherapy in Iran especially children population in Yazd is less known. In the present study, we investigated antibody titer against HBV infection in children with cancer. Our results showed that about 29% of the patients did not have protective antibody level in their serum. Protective antibody level was considered ≥ 10 mIU/ml. These findings were consistent with other published data (10, 12). Based on other study, reduction of protective serum antibody for Hepatitis B virus was affected by immunosuppressive effects of cytotoxic therapy more than diphtheria, tetanus, pertussis. It confirmed that chemotherapy and cytotxic drugs are toxic for lymphocytes which produce antibody. Other studies confirmed that administration of booster diphtheria, tetanus, pertussis vaccines after completing chemotherapy causes 100% of patients to produce protective antibody persistently (17). The finding shows that intensive chemotherapy does not entirely kill specific B cell memory and T cells memory, although the humoral immune responses in the patients are not undetectable in their serum. In conclusion, our study demonstrated that intensive chemotherapy leads to loss of protective Hepatitis B serum antibody titers in about 29% of the patients. According to other studies, booster vaccinations started at 6-month after stopping chemotherapy can be safely administered and effectively restore a sustained effect in humoral immunity against Hepatitis B infection. So we suggest further studies for determining optimal timing and number of booster doses of hepatitis B vaccines in children after chemotherapy treatment.

## Conclusion

Our findings demonstrate that immunity to Hepatitis B in children after chemotherapy is significantly lower than those in healthy control. Thus it is essential to design new studies for determining optimum time , dose and schedule for vaccination after chemotherapy in children. 
